# In-vitro Evaluation of Solution Pressurised Metered Dose Inhaler Sprays with Low-GWP Propellants

**DOI:** 10.1007/s11095-025-03830-6

**Published:** 2025-02-12

**Authors:** Daniel J. Duke, Lingzhe Rao, Benjamin Myatt, Phil Cocks, Stephen Stein, Nirmal Marasini, Hui Xin Ong, Paul Young

**Affiliations:** 1 Department of Mechanical & Aerospace Engineering, Monash University, Clayton, 3168 Victoria Australia; 2 Kindeva Drug Delivery, Loughborough, LE11 5RB United Kingdom; 3 Kindeva Drug Delivery, Woodbury, 55129 Minnesota United States of America; 4 Respiratory Technology, Woolcock Institute of Medical Research, Macquarie Park, 2113 New South Wales Australia; 5 Macquarie Medical School, Faculty of Medicine, Health & Human Sciences, Macquarie University, Sydney, 2109 New South Wales Australia; 6 Department of Marketing, Macquarie Business School, Macquarie University, Sydney, 2109 New South Wales Australia

**Keywords:** pMDI, low-GWP, propellant, HFA152a, HFO1234ze(E)

## Abstract

**Purpose:**

The transition from high Global Warming Potential (GWP) propellants such as HFA134a to low-GWP alternatives such as HFA152a and HFO1234ze(E) in pressurised metered dose inhalers (pMDIs) poses a number of challenges for inhaled pharmaceutical product development. Changes in chemicophysical properties will alter product performance, impacting *in-vitro* bioequivalence metrics. This study investigates those differences using equivalent pMDI hardware and formulations.

**Methods:**

Aerodynamic particle size distribution (APSD) measurements, laser diffraction and high-speed imaging were used to compare the performance of HFA134a, HFA152a and HFO1234ze(E) solution formulations of beclomethasone dipropionate. Propellant-only placebos, cosolvent-free solutions, and ethanol solutions at 8% and 15% w/w were investigated.

**Results:**

HFA152a formulations had increased drug deposition on the actuator and throat while HFO1234ze(E) produced comparable APSD performance to HFA134a formulations. Plumes from HFA152a formulations spread more rapidly and were less stable and repeatable than those from HFA134a. HFO1234ze(E) plumes spread more slowly than HFA134a, but converged with HFA134a ex-mouthpiece. Differences between propellants were moderated by the addition of ethanol.

**Conclusion:**

Plume stability is a driver of differences between formulations in the near-orifice region. Shot-to-shot repeatability differences are more pronounced ex-mouthpiece, where mixing with ambient air is dominant. Modifications to low-GWP pMDI product actuator orifice and mouthpiece geometries may provide a route to improved *in-vitro* product bioequivalence relative to current pMDIs. Differences between formulations are modest and may be managed through a combination of formulation, orifice and mouthpiece geometry changes. These generic formulations provide a database of benchmark data against which the performance of low-GWP products may be compared.

**Supplementary Information:**

The online version contains supplementary material available at 10.1007/s11095-025-03830-6.

## Introduction

Choice of propellant plays a major role in the performance of solution-based pressurised metered dose inhalers (pMDIs) [1, 2]. Propellant properties dictate the structure of the plume, the size of the droplets formed and the properties of the particles produced through a range of complex chemical, thermodynamic and fluid-mechanical interactions [3–5]. Due to the complexity of these processes, the mechanisms by which propellant properties affect the pharmaceutical performance of a product (i.e. fine particle fraction, droplet size, etc.) and thus drug delivery efficacy are not fully understood.

The impending transition from hydrofluoroalkane (HFA) propellants such as HFA134a and HFA227ea to low global warming potential (low-GWP) propellants such as HFA152a and HFO1234ze(E) will involve significant changes in formulation chemico-physical properties such as vapour pressure and density [6]. Lack of understanding of the influence of these properties on pharmaceutical product performance poses a challenge for the design of effective solution-based pMDIs [7–9].

**Table 1:** Relevant propellant chemicophysical properties and derived properties

Propellant	HFA134a	HFA152a	HFO1234ze(E)
Global warming potential	1430 kg CO$_2$e	124 kg CO$_2$e	7 kg CO$_2$e
Vapor pressure at 20$^{\circ }$C	5.72 bar	5.12 bar	4.27 bar
Saturation temperature at 1 atm.	-26.1$^{\circ }$C	-24.0$^{\circ }$C	-19.0$^{\circ }$C
Surface tension at 20$^{\circ }$C	8.7 mN/m	10.4 mN/m	9.6 mN/m
Liquid density	1207 kg/m$^{3}$	904 kg/m$^{3}$	1170 kg/m$^{3}$
Vapor density	5.24 kg/m$^{3}$	3.37 kg/m$^{3}$	5.69 kg/m$^{3}$
Jakob Number Eq. 1	0.41	0.32 *(-23%)*	0.36 *(-12%)*
Orifice Reynolds Number Eq. 3	$3.5 \times 10^4$	$3.2 \times 10^4$ *(-7%)*	$2.4 \times 10^4$ *(-30%)*
Orifice Weber Number Eq. 4	$13 \times 10^3$	$9.1 \times 10^3$ *(-31%)*	$9.0 \times 10^3$ *(-32%)*
Theoretical stable droplet size	5.7 µm	9.7 µm *(+70%)*	7.5 µm *(+32%)*
($$d^*$$, Eq. 6)			

Changes for low-GWP propellants are given as percentages relative to HFA134a

The chemico-physical properties of the propellant not only influence the solubility and stability of the active pharmaceutical ingredient (API). They also dictate the properties of the spray plume in the actuator near-orifice region [6, 10]. The overall internal geometry of the valve and actuator (such as the sump and valve stem volume, and ratio of metering valve to actuator spray orifice diameters) act together with the formulation, particularly the propellant vapour and liquid densities, to determine the amount of internal vapourisation that initiates aerodynamic atomisation in the nozzle orifice [11]. Propellant vapor pressure and orifice size subsequently dictate spray plume velocity and momentum which in turn controls the rate of droplet formation and mixing in the early regions of the spray (i.e. inside the mouthpiece) [12]. The surface tension of the formulation also drives initial droplet size [13]. The boiling point and latent heat of vapourisation of the propellant determine the degree to which the plume flash-evaporates when it exits the orifice; flashing is known to enhance atomisation and produce finer droplets [12]. All these factors influence the geometry of the spray plume and particle maturation [14, 15], which influences API throat deposition and fine particle fraction [3].

The chemico-physical properties of alternative low-GWP propellants are less conducive to the production of fine droplets and particles than current-generation propellants [6]. This is due to a combination of vapor pressure and density changes acting to reduce spray momentum, increased surface tension resulting in larger droplets, reduction in the sensible enthalpy (i.e. thermal energy) available, and an increase in the latent heat required to vapourise the propellant. Some key properties that demonstrate these changes are given in Table 1 (data from [16–18]).

Both HFA152a and HFO1234ze(E) have a reduced propensity to flash-evaporate when compared to HFA134a. This can be quantified by the Jakob number [19], the ratio of sensible to latent heat:

1 $$\begin{aligned} \textrm{Ja} = \frac{c_p \left( T_0 - T_{\textrm{sat}} \right) }{\Delta h_{fg}} \end{aligned}$$

In addition, while HFA152a has a higher vapor pressure than HFO1234ze(E), its liquid density is much lower [17]. This yields low spray momentum, which will likely cause more rapid mixing. HFO1234ze(E) has a similar liquid density to HFA134a but a lower vapor pressure [16], thus reducing spray velocity and reducing mixing, since for an ideal nozzle the velocity scales with pressure and density according to the following:

2 $$\begin{aligned} \overline{U} \propto \sqrt{ \frac{2 \left( P_{\textrm{sat}} - P_{\textrm{amb}} \right) }{\rho _{\textrm{mix}}}}. \end{aligned}$$

For both HFA152a and HFO1234ze(E) we also expect droplet sizes to increase due to higher surface tension (Table 1). The presence of cosolvent will likely moderate the differences between propellants, as has been previously observed in HFA134a, HFA152a and HFO1234ze(E) systems [3].

Water content is a critical factor in particle formation [20]. Aside from water solubility variations, the ability of the spray plume to entrain humid surrounding air into the spray core can influence particle maturation, residual droplet size and therefore region/location of deposition [15, 21]. Differences in turbulent mixing and entrainment between propellants can vary significantly from one shot to the next and occur at very short time and length scales due to high Reynolds and Weber numbers Eqs. 3-4. Typical values relevant to this study can be found in Table 1. These differences are not easily captured by conventional spray pattern and plume geometry methods [22].

3 $$\begin{aligned} \textrm{Re}_D = \frac{ \rho \overline{U} D }{ \mu }\end{aligned}$$

4 $$\begin{aligned} \textrm{We}_D = \frac{ \rho \overline{U}^2 D }{\sigma } \end{aligned}$$

**Table 2:** Formulation properties and experimental conditions

	**pMDI Formulations**			
API	*None*	*Beclomethasone Dipropionate (BDP)*		
Formulation	Propellant-only	Cosolvent-free	Ethanol-containing	
	Placebo	solution	solution formulations	
API concentration	0 mg/mL	0.05 mg/mL	2.0 mg/mL	2.0 mg/mL
Co-solvent	None	None	8% w/w EtOH	15% w/w EtOH
	**Operating conditions**			
Ambient temperature	21$^{\circ }$C	21$^{\circ }$C	21$^{\circ }$C	21$^{\circ }$C
Ambient humidity	50%	34%	50%	50%
Air flow rate	28.3 L/min	30.0 L/min	28.3 L/min	28.3 L/min
Sample size (APSD)	−	$$ n=24$$	$$n=5$$	$$n=5$$
		$$ N=3$$	$$N=3$$	$$N=3$$

At present there is insufficient data to determine how flash evaporation, turbulent mixing and their subsequent effects on particle properties will be affected by the shift to low GWP propellants. Although evaporation dynamics play a larger role in delivery efficiency than initial atomisation effects in drug delivery [23], it is the atomisation region in which the largest changes between propellants occurs, and this region is not well understood. Recent work by Wang et al [15] shows how propellant choice affects the acoustics and residual nonvolatile aerosol size distribution of placebo and suspension formulations. However, there is relatively little data available for solution formations [6, 9].

In this paper we have used a combination of conventional pharmacopoeial measurement approaches, laser droplet sizing and ultra high-speed plume imaging to investigate the role of low GWP propellants HFA152a and HFO1234ze(E) on solution-based pMDI formulations using conventional pMDI hardware across a range of cosolvent concentrations. We show how subtle changes in the structure of the plume in the near-orifice and ex-mouthpiece region relative to HFA134a controls are correlated with changes in droplet size and particle outcomes.

## Materials and Methods

### Materials

Twelve formulations were considered in this study, consisting of four API/cosolvent combinations in three propellants. These are summarised in Table 2. A standard set of pMDI hardware was used consistently across all formulations. The first set of formulations are propellant-only placebos of three propellants; HFA134a, HFA152a or HFO1234ze(E). The second set are low-dose solutions of 0.05 mg/mL beclomethasone dipropionate (BDP) dissolved in propellant only as per Stein et al [3]. These were used to model aerodynamic particle size distribution outcomes in the limit of no cosolvent where propellant differences are expected to be largest. The third and fourth sets are conventional solution formulations of 2 mg/mL BDP in ethanol at 8% and 15% w/w respectively, in each of the three propellants. This large set of formulations permits consideration of the effects of low-GWP propellants on cosolvent-free formulations and observation of the moderating effect of cosolvent addition up to a typical concentration range. BDP was supplied by Kindeva Drug Delivery (Loughborough, United Kingdom). Methanol, anhydrous ethanol and other high-performance liquid chromatography (HPLC) grade solvents were supplied by Sigma-Aldrich (Castle Hill, New South Wales, Australia). Industrial-grade propellants (99.5% purity) were supplied by A-Gas Australia.

A standard Kindeva pMDI actuator with 0.3 mm orifice diameter, 0.8 mm orifice length and 14 mm$^3$ sump volume was used for all the droplet sizing and imaging experiments. Propellant placebos, 8% and 15% ethanol containing solutions were prepared at Monash University. Micronised BDP was weighed using a precision balance (AS 62.R2+, Radwag) and adding ethanol to form a solution concentrate which was homogenised and dispensed into 16 mL FEP-coated aluminium canisters (Kindeva). A 50 µL metering valve (BK357, Bespak, UK) was crimped to the canister using a Pamasol laboratory plant (2002, Pamasol, Switzerland) and the canisters were filled with propellant to 200 nominal actuations.

**Fig. 1:**
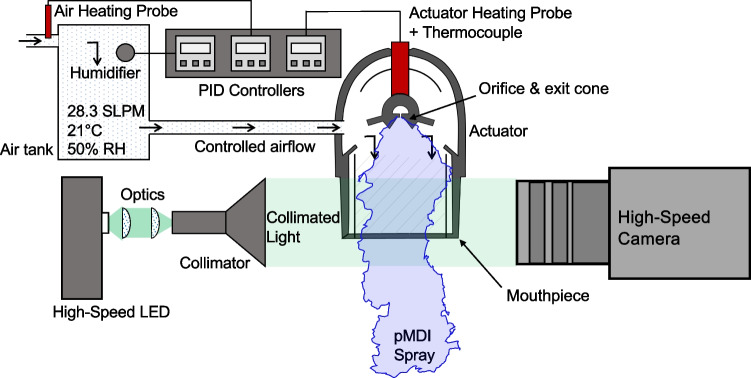
Simplified schematic of the high-speed imaging system, viewed from above.

The cosolvent-free BDP solutions were prepared at Kindeva’s facility in Loughborough (UK) by addition of BDP by weight directly to the canister, prior to valve crimping and pressure filling with propellant. Canisters were then sonicated for a minimum of 60 minutes to aid dissolution of the BDP in the liquified propellant. A Kindeva actuator with 0.65 mm orifice length was used for the cosolvent-free formulations. The concentration of 0.05 mg/mL was chosen based on saturated solubility data of BDP in each propellant collected prior to preparing these formulations.

### Cascade Impaction APSD Measurements

For the 2 mg/mL BDP 8% and 15% w/w ethanol solution formulations, an Andersen cascade impactor (ACI) with USP-induction port (“throat”) was used to assess the aerosol performance and pulmonary deposition profiles of the pMDI formulations as per United States Pharmacopoeia (USP) Chapter $<601>$ [24]. The measurements were performed at the Woolcock Institute (Sydney, Australia). For the 0.05 mg/mL cosolvent-free BDP formulations, testing was conducted at Kindeva Drug Delivery (Loughborough, UK) using a Next Generation Impactor (NGI, MSP Corporation, USA) operating at 30 L/min via a USP-induction port. The Fine Particle Dose (mg) was defined as the mass of drug particles with aerodynamic diameters less than 5.0 microns and the Fine Particle Fraction (%) is the Fine Particle Fraction (mg) divided by the measured ex-actuator Delivered Dose (mg).

ACI plates and NGI cups were coated prior to testing to ensure particle bounce was minimised. Multiple actuations were fired into the cascade impactor apparatus with a 30 second delay time between actuations. This is longer than the experimentally-determined minimum time required for the pMDI to thermally equilibrate [10]. Samples were collected from the actuator, throat, coupler, NGI stages and filter and analysed for drug content. A suitable solvent was used to recover the deposited API, and subsequent test samples were then quantified using reverse phase HPLC with ultraviolet detection, against calibrated standards. For the ACI measurements and all subsequent optical measurements, an average relative humidity of 50% was maintained Table 2. The NGI measurements were performed at an average relative humidity of 34%.

Data were processed using Copley Inhaler Testing Data Analysis Software (CITDAS, Copley Scientific, UK). Additional details are given in Table 2 and further detail may be found in Duke et al [6]. The differences among groups were determined using one-way or two-way analysis of variance (ANOVA) followed by posthoc Tukey tests using GraphPad Prism 9. The difference was considered significant if the p-value was $\le 0.05$.

### Laser Diffraction Droplet Size Measurement

A volume-based particle size distribution was measured by laser diffraction using a Spraytec apparatus (Malvern Instruments, Worcestershire, UK) [25]. Since the role of propellant on droplet size distribution is likely to be more pronounced closer to the actuator orifice, the measurements were taken immediately post-mouthpiece with the laser beam aligned to the centre of the mouthpiece. Before testing, five shots from each canister were fired to waste to ensure adequate valve priming. The droplets were measured with a 300 mm lens using a dispersion refractive index 1, particle refractive index 1.56, estimated density of 1.3 g/cm$^3$ and data acquisition rate of 10 kHz. Data from $n=25$ repeated measurements were obtained for each formulation.

Droplet size statistics were calculated during the steady period of the spray which was defined as the time where the laser absorption exceeded the half-maximum of the peak value, with a cut-off filter applied at 46 µm. The laser absorption threshold is selected to ensure consistency across measurement techniques and maintain a minimum signal-to-noise ratio.

**Fig. 2:**
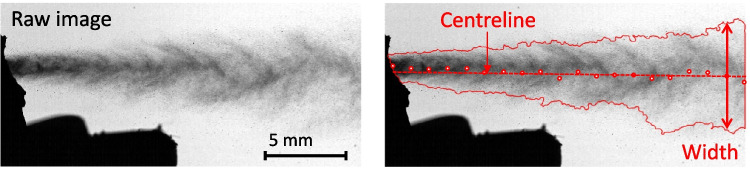
Sample plume image (left) and definition of spray width (right).

### High Speed Plume Imaging

In order to capture rapid temporal variations at small scales in the spray plume, a custom high-speed imaging system using diffuse backlit illumination was developed at Monash University [6]. A simplified diagram of this facility is shown in Fig. 1. A high speed camera (SA-Z, Photron, USA), 150 mm macro lens (Nikon, Japan) and custom pulsed LED system [26] (Monash University) with beam expander/collimator (Edmund Optics, Singapore) was placed either side of the mouthpiece to capture the structure of the plume with an effective exposure time of 350 ns at speeds of 20,000 to 140,000 frames/s with a fixed magnification of 31 µm/pixel.

Owing to the need to have an unobstructed view of the mouthpiece region, inhalation was simulated using an air conditioning system which delivered a constant flow of 28.3 standard L/min at 50% relative humidity and 21$^\circ$C with positive pressure applied to the top of the actuator via a 3D printed adaptor. The adaptor was also fitted with a pneumatically operated plunger which depressed the pMDI unit to trigger the spray with repeatable timing in synchronisation with the image capture. In order to prevent cooling and ice formation around the actuator due to evaporative self-cooling of the formulation with repeated, frequent actuations, a 15 W heating element was installed behind the nozzle block. The heater was used with a thermocouple and PID temperature controller to obtain constant nozzle block temperature throughout all tests. The timing between spray events was fixed at 25 s in order to minimise thermal effects.

The presence of the actuator mouthpiece and orifice exit cone impedes optical access to the orifice exit, where most primary droplet formation occurs [5]. However, removal of the mouthpiece can alter the plume structure as the mouthpiece constrains the periphery of the plume. To overcome this impediment, all experiments were repeated with three imaging configurations:

- Ex-orifice condition: Mouthpiece removed and nozzle block machined back to partially remove the exit cone, permitting direct observation of the orifice exit plane ($N=60$ repeats of $n\approx 2000$ time-resolved snapshots per formulation).
- Near-orifice condition: Mouthpiece removed but nozzle block unmodified ($N=20$ repeats of $n\approx 6000$ time-resolved snapshots per formulation).
- Ex-mouthpiece condition: Sides of the mouthpiece removed, but the top and bottom left intact to preserve vertical constraint on the plume development ($N=15$ repeats of $n\approx 14000$ time-resolved snapshots per formulation).

The same actuator was used for all formulations across each region of interest in order to avoid bias in the results due to actuator variability. Prior to use, these actuators were compared against two other randomly selected actuators (and an unmodified production actuator) to ensure that the spray plume was qualitatively similar and that the selected actuators did not exhibit any atypical behaviour. In total, 10 TB of imaging data was obtained during the study. An example high-speed image of an ex-orifice HFA134a plume with the mouthpiece removed is shown in Fig. 2 (left). A custom algorithm was developed in MATLAB (Mathworks) to detect the spray boundary and centerline in each image at multiple streamwise locations, as per Fig. 2 (right). A spray width profile was developed for each shot as a function of both time and distance from the orifice.

The spray width in each image at each position varies due to turbulent mixing and instabilities at the plume boundary, so the spray width data was ensemble-averaged over many repeated shots and time-averaged over the steady period of the spray to obtain a time-average, ensemble-average mean and standard deviation width profile for each formulation. The steady period of the spray was defined by the product of the coefficients of variance of the spray inclination angle and the volume-integrated light extinction in a region adjacent to the orifice. A mathematical definition is given in the Appendix. All imaging results are conditionally averaged on this product exceeding the half maximum value, to ensure consistency between the laser diffraction and imaging studies.

**Table 3:** Aerodynamic particle size distribution results for all propellant and formulation combinations tested

Propellant	HFA134a	HFA152a	HFO1234ze(E)
	*0.05 mg/mL BDP in Propellant only*		
Total Dose Per Shot [µg]	2.73 ± 0.044	2.77 ± 0.069	2.01 ± 0.048 **
Delivered Dose [µg]	2.13 ± 0.016	2.25 ± 0.069	1.66 ± 0.042 **
Fine Particle Dose [µg]	1.89 ± 0.016	1.59 ± 0.06 *	1.43 ± 0.037 **
Fine Particle Fraction [%]	88.5 ± 2.02	70.7 ± 1.55 **	85.9 ± 0.11
MMAD, GSD	N/A	N/A	N/A
Actuator+coupler+throat deposition	30.16 ± 1.51	39.80 ± 1.11 **	28.23 ± 0.16
[% of total API recovered]			
	*2.0 mg/mL BDP in 8% w/w Ethanol*		
Total Dose Per Shot [µg]	99.57 ± 5.09	97.18 ± 2.33	100.80 ± 3.40
Delivered Dose [µg]	84.86 ± 1.70	81.21 ±0.40 *	88.34 ±3.43 *
Fine Particle Dose [µg]	47.24 ± 0.50	41.62 ± 2.13 *	53.55 ± 1.38 **
Fine Particle Fraction [%]	55.69±1.67	51.26 ±2.83	60.67 ±2.66
MMAD [µm]	1.16 ± 0.00	1.26 ±0.05	1.29 ±0.05
GSD [µm]	1.74 ± 0.10	1.78 ±0.01	1.75 ±0.02
Actuator+coupler+throat deposition	51.7 ± 1.96	64.7 ± 2.14*	52.6 ± 1.81
[% of total API recovered]			
	*2.0 mg/mL BDP in 15% w/w Ethanol*		
Total Dose Per Shot [µg]	97.19 ± 2.60	101.97 ± 2.78	103.00 ± 5.87
Delivered Dose [µg]	85.07 ± 1.90	86.42 ± 2.03	89.43 ± 3.64*
Fine Particle Dose [µg]	33.49 ± 2.69	33.70 ± 2.86	36.94 ± 5.73
Fine Particle Fraction [%]	39.34±2.49	38.97 ±2.94	41.19 ±4.94
MMAD [µm]	1.34 ± 0.05	1.40 ±0.03	1.49 ±0.10
GSD [µm]	2.03 ± 0.07	2.00 ±0.02	1.99 ±0.03
Actuator+coupler+throat deposition	63.5 ± 2.57	64.7 ± 2.31	61.3 ± 4.71
[% of total API recovered]			

Significance indicators: * $$p<0.05$$, ** $$p<0.01$$

In addition to plume width, light extinction in the spray provides insight into the scattering surface area from the droplets, which depends on both their size and number density [27]. Changes in scattering were measured by calculating a volume-averaged spray width profile. The measured light extinction was first normalized by the incident intensity to remove the influence of the background brightness, and the resulting profiles were then radially integrated across the measured width of the plume at each streamwise position (*x*) to obtain a volumetric extinction integral, defined as:

5 $$\begin{aligned} \overline{I_{V}}(x) = \frac{1}{t_1-t_0} \int _{t_0}^{t_1} \int _{r_0(x)-r(x)}^{r_0(x)+r(x)} I \, \textrm{d} r \, \textrm{d} t \end{aligned}$$

Plume width was defined by the spray radius *r* about its centerline $r_0$ during the steady period of the spray ($t_0$ to $t_1$).

## Results and Discussion

### Impactor measurements

**Fig. 3:**
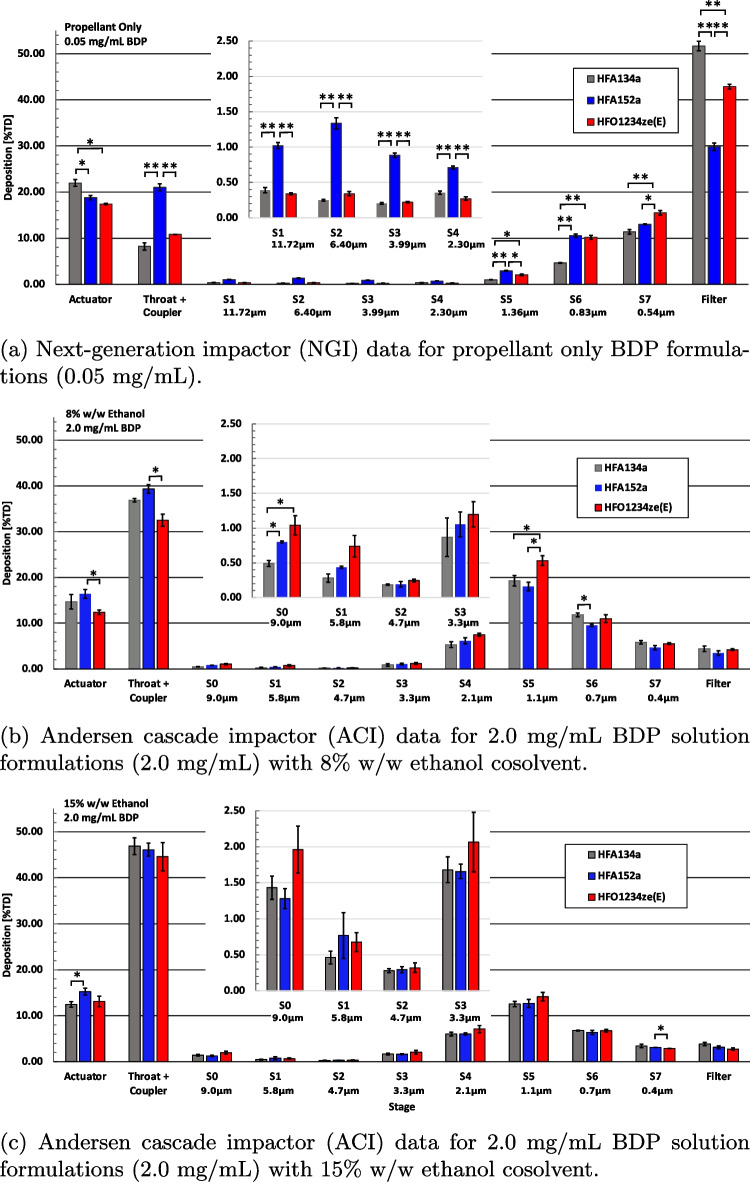
Aerodynamic particle size distribution results for BDP formulations. The vertical axis is given as a percentage of total dose, ex-valve. Significance indicators: * $p < 0.05$, ** $p < 0.01$.

APSD measurements via ACI (2 mg/mL BDP, 8% and 15% w/w ethanol) and NGI (0.05 mg/mL BDP, 0% ethanol) are shown in Table 3. Corresponding aerodynamic particle size distributions are shown in Fig. 3 for 0.05 mg/mL BDP (Fig. 3a), 2 mg/mL BDP with 8% w/w ethanol (Fig. 3b) and 2 mg/mL BDP with 15% w/w ethanol formulations (Fig. 3c). Differences in the delivery efficiency (e.g. fine particle fraction) for the different propellants are largest for the cosolvent-free formulations (Fig. 3a). The addition of ethanol moderates the differences in propellant properties and reduces the statistical significance of the differences in APSD between formulations [3]. A direct comparison with the ethanol-containing 2.0 mg/mL BDP solution formulations and the cosolvent-free formulation is not possible due to the differences in test protocol used (NGI vs ACI) and the difference in relative humidity (Table 2). However, protocols and test conditions were held constant for the testing of formulations of the three propellants, permitting a relative assessment of APSD performance for each set of experiments.

Differences in delivery efficiency between propellant formulations were most notable for the ethanol-free 0.05mg/mL solution formulations with the HFA152a formulation having fine particle fractions 17.8% and 2.5% lower than the HFA134a and HFO1234ze(E) formulations Table 3, respectively. The HFA152a formulation had noticeably higher API deposition on the USP inlet (“Throat + Coupler”) and on the upper stages of the NGI compared to the other formulations. Increased API deposition on the USP inlet is influenced by the size of the atomized droplets [23], the dynamics of the plume [6] as well as the rate of evaporation of the atomized droplets.

The elevated deposition for the HFA152a cosolvent-free formulation on the upper stages of the impactor is likely indicative that a portion of the atomized droplets have not fully evaporated prior to reaching the NGI [28] and that the HFA152a formulation droplets evaporate more slowly than HFA134a or HFO1234ze(E) formulation droplets. Interactions between precipitation and evaporation rates are complex and require further investigation. The fine particle dose (mg) was lowest for the HFO1234ze(E) formulation, but this is due to the total dose per actuation being lower for this formulation and does not indicate decreased delivery efficiency.

**Fig. 4:**
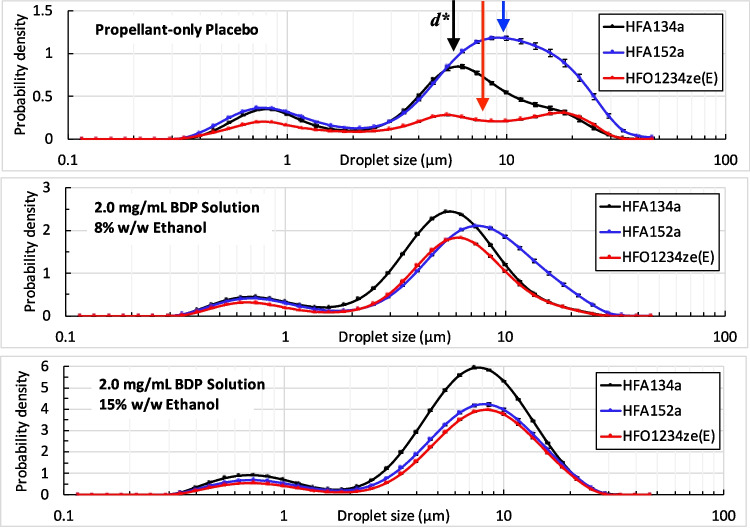
Laser droplet sizing data from Malvern Spraytec for propellant-only placebo formulations (top), 8% and 15% w/w BDP solutions (middle, bottom). The vertical arrows represent the theoretical stable droplet size $d^*$ Eq. 6.

APSD results for the ethanol-containing 2.0 mg/mL BDP solution formulations (Fig. 3b and c) are relatively similar for all propellants, with the differences being reduced at higher cosolvent fractions. Table 3 shows how the differences in APSD performance between the different propellant systems are reduced with increasing ethanol concentration.

The difference in fine particle fraction between propellants was less noticeable for the formulations with 8% ethanol (HFA152a formulation was 8% and 16% lower than HFA134a and HFO1234ze(E) formulations, respectively) and 15% ethanol (HFA152a formulation was 1% and 5% lower than HFA134a and HFO1234ze(E) formulations, respectively). API deposition on the actuator, coupler and throat was found to be $25\% \pm 2\%$ higher for HFA152a than for HFA134a in both the cosolvent-free and 8% w/w ethanol formulations. This effect reduces at the higher cosolvent fraction. No such change in API deposition was noted for HFO1234ze(E) formulations under any condition.

HFA152a and HFO1234ze(E) formulations with ethanol showed increased mass median aerodynamic diameter (MMAD) relative to HFA134a formulations, in the order of 9-11% ($\pm 1\%$), as per Table 3. MMAD could not be determined for the cosolvent-free 0.05mg/mL BDP formulations since such a high proportion of the API was collected on the filter . The fundamental propellant properties of both HFA152a and HFO1234ze(E) lead to larger initial droplet size and reduced mixing which support an increased MMAD, relative to HFA134a formulations. The significance of these results is that a change in either fundamental droplet atomisation physics or spray plume geometry will need to be affected if a match in APSD between HFA134a and low-GWP solution formulations is desired. This could arise through either changes to the actuator or formulation chemicophysical properties. In the subsequent sections, both routes (droplet properties and plume geometry) are investigated.

**Table 4:** Ex-mouthpiece droplet size statistics as determined by laser diffraction

Propellant	HFA134a	HFA152a	HFO1234ze(E)
	*Propellant-only placebo*		
Sauter Mean Diameter $$d_{32} $$ [µm]	15.58 ± 0.56	19.45 ± 0.39	18.85 ± 0.22
Droplet MMAD [µm]	17.36 ± 0.63	20.90 ± 0.41	19.87 ± 0.18
Droplet GSD [µm]	1.57 ± 0.08	1.48 ± 0.06	1.34 ± 0.03
	*2.0 mg/mL BDP in 8% w/w Ethanol*		
Sauter Mean Diameter $d_{32}$ [µm]	11.00 ± 0.09	14.76 ± 0.22	11.40 ± 0.11
Droplet MMAD [µm]	11.99 ± 0.12	16.04 ± 0.24	12.30 ± 0.14
Droplet GSD [µm]	1.68 ± 0.02	1.52 ± 0.02	1.62 ± 0.03
	*2.0 mg/mL BDP in 15% w/w Ethanol*		
Sauter Mean Diameter $d_{32}$ [µm]	13.18 ± 0.11	13.82 ± 0.07	13.63 ± 0.04
Droplet MMAD [µm]	13.95 ± 0.12	14.69 ± 0.08	14.40 ± 0.04
Droplet GSD [µm]	1.50 ± 0.02	1.47 ± 0.01	1.46 ± 0.01

### Droplet Sizing

To understand the causal factors that contribute to the trends observed in the APSD data with respect to particle size and delivered dose, measurements of droplet properties closer to the actuator orifice are required. To this end, ex-mouthpiece droplet size distributions were obtained using laser diffraction for the propellant-only placebo, and 2.0 mg/mL BDP solutions with 8% and 15% w/w ethanol. Time-average, ensemble average normalized probability densities for droplet size are shown in Fig. 4. These data are averaged during the steady period of the spray (conditional on laser absorption exceeding half the maximum value). A quantitative comparison of the size distribution data is given in Table 4. In all configurations, droplet size increases relative to the HFA134a control case, with the addition of ethanol in the formulation having a moderating effect on droplet size. These changes can mostly be explained by changes in formulation liquid density $\rho$ and surface tension $\sigma$ which define a minimum stable droplet size $d^*$ for a given spray. The value of $d^*$ can be estimated from the critical Weber number [29]:

6 $$\begin{aligned} \textrm{We}^* = \frac{\rho _v \overline{U}^2 d^*}{\sigma } \approx 1 \end{aligned}$$

Indicative values of $d^*$ for propellant-only placebos are given in Table 1. The theoretical and observed droplet sizes are best compared by considering the equivalent average diameter of spherical droplets having the same volume to surface area ratio (*V*/*S*) as that recorded in the spray with probability distribution *q*(*d*) [30]. This is described by the Sauter mean diameter ($d_{32}$) which is defined as:

7 $$\begin{aligned} d_{32} = \frac{6V}{S} = \frac{\int d^3 q(d) \, \textrm{d} d}{\int d^2 q(d) \, \textrm{d} d} \end{aligned}$$

The correlation between predicted droplet size from the chemicophysical properties only and the measured droplet size is $R^2 = 0.81$ for $d_{32}$ and $R^2 = 0.86$ for droplet MMAD, with $d_{32}$ being approximately double the value of $d^*$. For the solution formulations, much but not all of the change in droplet size (Table 4) is driven by chemicophysical property changes. The significance of this result is that manipulation of the droplet and formulation properties may be insufficient to achieve comparable APSD outcomes between HFA134a and low-GWP solution formulations.

### High speed plume imaging - time average statistics

**Fig. 5:**
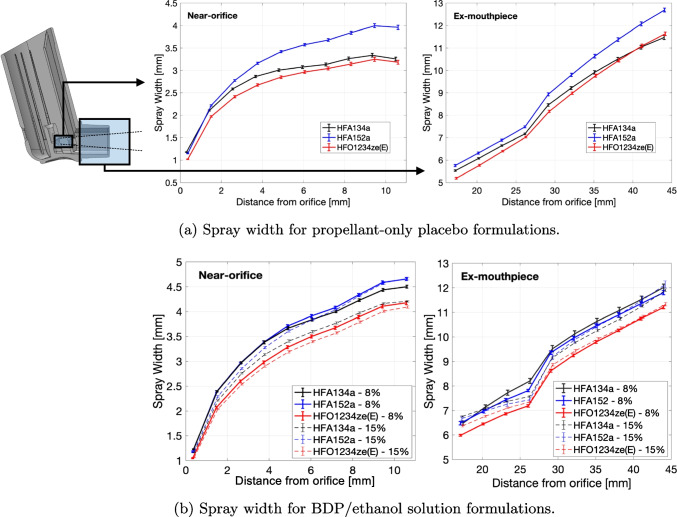
Spray width near-orifice (left) and ex-mouthpiece (right). Error bars indicate a combined standard deviation including both temporal and shot-to-shot variations.

While droplet size trends can be explained through consideration of formulation properties, changes in APSD outcomes with propellant are more complex. Unsteady variations in plume geometry and mixing are expected to play a role. To investigate this, high speed imaging was used to measure the transient width, angle and intensity of the spray plume as described in Section “High Speed Plume Imaging - Stability and Repeatability”. Calculated spray width profiles are shown for all formulations in Fig. 5. Measurements were taken both near-orifice (left) and ex-mouthpiece (right). The propellant-only placebos Fig. 5a show HFA152a having a wider plume and HFO1234ze(E) a narrower plume relative to that of HFA134a. This is consistently observed from  1 mm from the orifice (i.e.  3 orifice diameters), and extends throughout the domain. The shape of the average width profiles are comparable for all cases. The sudden jump in spray width in Fig. 5 from 25-30 mm is caused by the flow exiting the mouthpiece, at which point it is no longer constrained in the transverse axis and expands quickly to equilibrium with the ambient pressure. As ethanol is added to the formulation Fig. 5b the differences in plume width between propellant systems reduce but follow the same trend as the propellant-only placebos.

**Table 5:** Ex-mouthpiece spray inclination and cone angles

Propellant	HFA134a	HFA152a	HFO1234ze(E)
	*BDP 2 mg/mL in 8% w/w ethanol*		
Spray inclination angle [deg.]	$-3.16 \pm 0.04 ^\circ$	$-4.19 \pm 0.06 ^\circ$	$-5.11 \pm 0.07 ^\circ$
Spray cone angle [deg.]	$22.12 \pm 0.50 ^\circ$	$21.68 \pm 0.41 ^\circ$	$19.79 \pm 0.30 ^\circ$
	*BDP 2 mg/mL in 15% w/w ethanol*		
Spray inclination angle [deg.]	$-5.59 \pm 0.09 ^\circ$	$-5.65 \pm 0.08 ^\circ$	$-5.17 \pm 0.07 ^\circ$
Spray cone angle [deg.]	$21.42 \pm 0.25 ^\circ$	$21.25 \pm 0.40 ^\circ$	$20.77 \pm 0.30 ^\circ$

Uncertainties are quoted at one sample standard deviation for $N=60$ replicates

From the spray width data, spray inclination angles were calculated with respect to the centerline axis of the mouthpiece by fitting a line of best fit to the peak extinction values, and then temporally averaging over the steady period of the spray and ensemble-averaging these results over repeated actuations. Spray cone angles were also calculated by projecting the spray width back to the orifice and measuring the angle averaged over ten streamwise stations across the field of view. These data are also then temporally and ensemble-averaged. The spray centerline inclination angle and cone angle data are shown in Table 5. We find that HFO1234ze(E) sprays are narrower, while HFA152a and HFA134a sprays have similar cone angles. HFO1234ze(E) sprays have slightly more negative (downward) inclination angles than HFA134a at low ethanol concentration, with little difference at higher ethanol concentration. Small variations of several degrees in cone and inclination angles can result in substantial changes in spray width at the throat and coupler, which may affect APSD outcomes. Small adjustments on the order of 0.1mm in the orifice design can exploit this sensitivity to adjust the spray plume geometry post-mouthpiece [31]. However, the effect of plume density must be considered in addition to the cone angle.

**Fig. 6:**
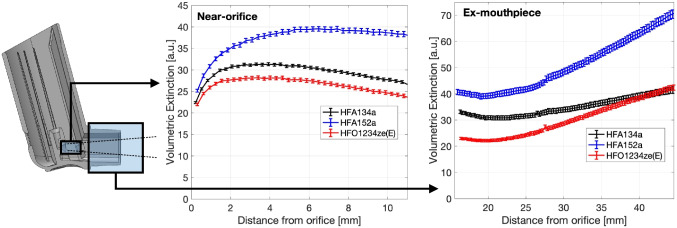
Volumetric extinction profiles $\left<\overline{I}_V\right>(x)$ for propellant-only placebos. Error bars indicate a combined standard deviation including both temporal and shot-to-shot variation. Results for BDP/ethanol solutions available in supplementary material.

Volume-integrated plume width Eq. 5 was also ensemble-averaged over many repeated shots to obtain an ensemble-average, time-average volumetric extinction $\left<\overline{I_V}\right>(x)$, which is shown in Fig. 6. The overbar denotes time averaging, and the angle bracket denotes ensemble averaging (from shot-to-shot). HFA152a has a consistently higher volumetric extinction relative to HFA134a, and HFO1234ze(E) has a consistently lower value relative to HFA134a. The spatial development of the volumetric extinction is similar across all propellants. The HFA152a profile diverges positively in the ex-mouthpiece region. Given that the cone angle remains relatively similar Table 5, the increase in volumetric extinction indicates a reduction in spray density, and a broader distribution of droplets throughout the plume which scatters light more effectively. Due to the nonlinear relationship between scattering and droplet number density, $\left<\overline{I_V}\right>(x)$ will increase as the spray expands until evaporation acts to reduce it. HFO1234ze(E) volumetric extinction is lower than the HFA134a values in the near-orifice region and converges with the HFA134a profile ex-mouthpiece, indicating a narrower initial spray that is diluted by mixing with the ambient air further downstream; this correlates with the reduced cone angle data in Table 5.

The differences in volumetric extinction and spray cone angle can be explained by differences in the chemicophysical properties of the propellants. Near-orifice spray structure is controlled by initial flash-evaporation of the superheated propellant [32], while behaviour further downstream of this region is dominated by mixing with the ambient air [33]. Expansion of vapour bubbles in the plume during flashing causes spray width to increase rapidly near the orifice. Initial divergence of the spray width and volumetric density should depend inversely on the vapour phase density at atmospheric pressure; bubbles inside the plume will expand to greater size if gas phase density is lower. Surface tension plays relatively little role in this region. Figures 5 and 6 support this hypothesis; initial divergence between formulations (Fig. 5a) correlate inversely with the vapour density (Table 1). HFA152a has a 36% lower vapour density relative to HFA134a, and the plume is observed to expand to a correspondingly larger ratio. HFO1234ze(E) has a 9% higher vapour density relative to HFA134a, and its plume expands by a correspondingly lesser amount. This effect explains the differences in the near-orifice behaviour of HFA152a in particular.

**Fig. 7:**
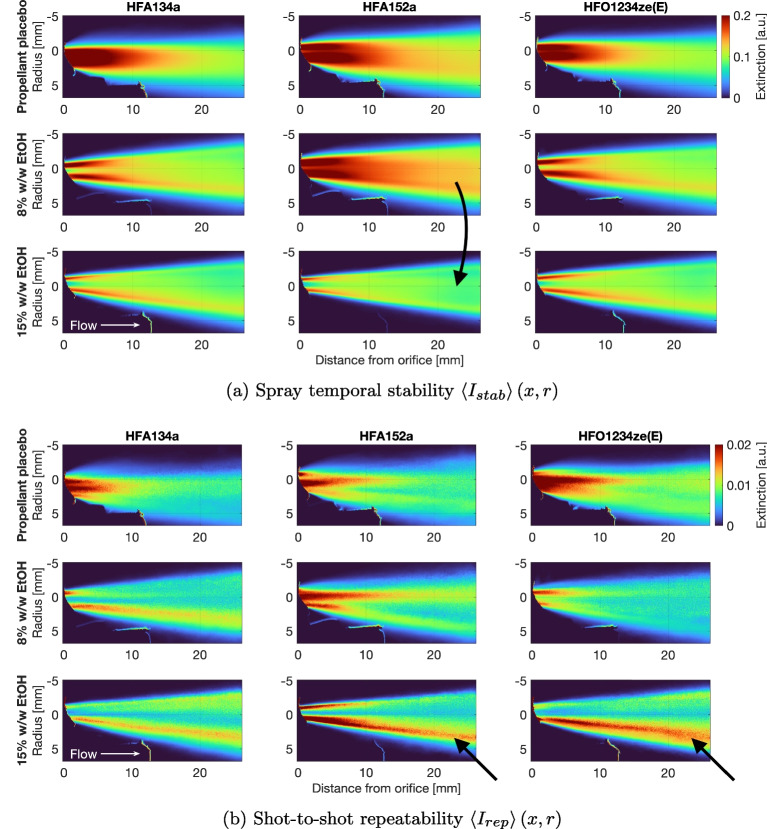
Spatial structure of plumes for all formulations in the near-orifice region, showing (a) stability Eq. A6 and (b) repeatability Eq. A7. Flow is left-to-right. The horizontal axes are distance from orifice (*x*) and vertical axes are radius (*r*), in mm. These data represent the average of $N=20$ actuations with $n \approx 6000$ snapshots per actuation.

Further downstream in the ex-mouthpiece region (Fig. 5b), it is the equilibrium liquid-vapour mixture density rather than the vapour density that determines mixing. Here, vapour expansion is complete, the droplet field is dilute [34] and the plume behaves like a dense turbulent jet [33]. HFA152a has a 17% reduction in mixture density relative to HFA134a under isenthalpic conditions. The spray should thus expand and decelerate by a corresponding amount; this agrees with the volumetric extinction profile in Fig. 6. Conversely, HFO1234ze(E) has a 2% increase in mixture density relative to HFA134a, so the far-field plume structure should be similar. As expected, we see HFO1234ze(E) and HFA134a plumes converge to a similar volumetric extinction after the mouthpiece in Fig. 6. The significance of these results is that they provide a clear explanation for why HFA152a formulations produce different outcomes to HFA134a and HFO1234ze(E) formulations. Given the differences in plume volumetric extinction and width, careful adjustment of the actuator orifice and mouthpiece are required. However, the data do not yet fully explain the differences between HFA134a and HFO1234ze(E) formulations; these are investigated in the next section.

### High Speed Plume Imaging - Stability and Repeatability

So far, we have considered only average plume behaviours. Here we investigate how plume structure varies statistically over time and from shot-to-shot. Here, time-averaging is denoted with an overbar and ensemble-averaging with angle brackets. Definitions and equations can be found in the Appendix. The time-average, ensemble-average plume structures ($\langle \overline{I} \rangle$, Eq. A5) are unremarkable, and do not exhibit any large differences between the different propellant based formulations beyond those shown in Figs. 5 and 6. These can be found in the supplementary material.

The temporal stability $\langle I_{stab} \rangle$ of the plume is defined as the standard deviation in time of the ensemble-average intensity Eq. A6, and is shown in Fig. 7a. These data represent the average of $N=20$ actuations from each pMDI unit with $n \approx 6000$ snapshots per actuation. Here, large values (red) indicate large temporal fluctuations and small values (blue) indicate little change over time. Adding ethanol to the propellant/formulation has the effect of stabilising the spray in time, which is evident by the reduction in red regions in the core of the spray in the lower rows of images. However, far more ethanol is required to achieve the same effect for HFA152a than for the other propellants. At 8% w/w ethanol, HFA134a and HFO1234ze(E) show significant reduction in temporal variability. HFA152a requires the addition of 15% w/w ethanol to the formulation to achieve a reduction to a comparable level of temporal variability across the propellant formulations. This is indicated by the arrow in Fig. 7a.

The shot-to-shot repeatability $\langle I_{rep} \rangle (x,r)$ of the plume is defined as the standard deviation across many measurements of the time-average intensity Eq. A7. This is shown in the near-orifice region in Fig. 7b. High values (red) indicate large variations from one spray to the next, with low values (blue) indicating little variation. Both HFA152a and HFO1234ze(E) sprays show greater shot-to-shot variation than HFA134a. The effect does not diminish with increasing ethanol content, but instead migrates from the core to the edges of the plume (indicated by arrows). These data provide some new insight into why HFO1234ze(E) formulations in particular yield different APSD outcomes to HFA134a formulations; the higher density of HFO1234ze(E) with respect to HFA134a leads to changes in the temporal unsteadiness and repetability of the plume. Adjustment of formulation chemicophysical properties (i.e. via altered ethanol concentration) can influence these differences.

## Conclusions

A combination of traditional aerodynamic particle size distribution measurements, droplet size distribution data and novel high-speed imaging measurements have been used to elucidate the differences between solution formulations containing HFA134a, HFA152a and HFO1234ze(E) propellants with matched hardware across various cosolvent levels.

HFA152a BDP solution formulations with ethanol had reduced FPF and increased actuator, coupler and throat deposition. When compared to HFA134a, all HFA152a formulations produced larger residual particle MMADs from APSD testing and larger MMADs of the atomized droplets from laser diffraction testing. They were less stable and had greater shot-to-shot variability than equivalent HFA134a sprays. These increases are attributable to formulation physicochemical property changes which give rise to larger initial droplet sizes. The lower density of HFA152a relative to HFA134a gives rise to greater expansion of the vapour phase and a wider, less stable spray. This helps explain the observed increases in API deposition on the actuator, coupler and throat and the corresponding reduction in FPF and FPD. We found that these changes are moderated with the addition of ethanol to the formulations, however HFA152a requires more ethanol to achieve the same degree of stabilisation as HFA134a sprays. The significance of these findings is that HFA152a sprays are subject to higher mouthpiece and actuator deposition. Where equivalent outcomes are required, actuator orifice adjustments are an effective means of adjusting performance [31]. Adjustment of the formulation (i.e. co-solvent concentration) may also be beneficial.

HFO1234ze(E) sprays were found to be consistently narrower than HFA134a sprays, and less stable under all conditions. Addition of ethanol to the formulations did not eliminate the differences. Near-orifice effects were found to correlate with vapour density due to flashing. Ex-mouthpiece effects correlated with formulation equilibrium mixture density due to mixing. The narrow, less stable sprays produced by HFO1234ze(E) formulations are less likely to interact with the actuator mouthpiece. The significance of these findings is that for the HFO1234ze(E) formulations, adjustment of the formulation (i.e. changing the co-solvent concentration to lower the mixture density) should be considered as a route to adjusting performance. Adjustment of the actuator orifice is a useful secondary route [31].

The practical implications of this study are that the factors driving the differences between HFA134a and HFA152a sprays are different to those driving differences between HFA134a and HFO1234ze(E) sprays. The former produces wider sprays and the latter narrower ones. Both are less stable than HFA134a sprays but in different ways. As such, different design approaches may be required depending on the choice of propellant. A range of pMDI hardware options may also need to be considered for various applications when adopting alternative low-GWP propellants in the development of pMDI products. From a plume structure and performance perspective the differences between propellants may be managed through a combination of formulation, actuator orifice and mouthpiece geometry changes. Adopting this approach holds promise for the generation of further supportive *in-vitro* data to aid the rapid development of low-GWP replacement pMDIs as part of a weight-of-evidence based approach [9, 35].

**Supplementary Information** Supplementary figures and data are uploaded with the submission and may be found on the Publisher’s web site.

## Supplementary Information

Below is the link to the electronic supplementary material.

Supplementary file 1 (pdf 2000 KB)

## Data Availability

The datasets generated during and/or analysed in this study are available from the corresponding author on reasonable request.

## Appendix A: Statistical definitions

The steady period of the spray was defined by the product of the coefficients of variance of the spray inclination angle and the volume-integrated light extinction in a region adjacent to the orifice;

A1 $$\begin{aligned} CV(t) = \frac{ \left<\theta (t)\right> \left<{I_V}(t)\right>}{ \sigma _\theta (t) \sigma _{I_V}(t)} \end{aligned}$$

where $\sigma$ is the sample standard deviation, and ${I_V}(t)$ is a transverse integral defined in Eq. 5. The steady spray period is defined as the time where $CV(t) < 3 \min (s(t))$.

The spray inclination angle $\theta (t)$ is calculated for each frame by finding the spray boundary using a threshold (Fig. 2 right panel), identifying the centerline, fitting a straight line $y(x,t)=m_c(t) x + c_c(t)$ to the centerline points using least-squares regression, and calculating the angle of the centerline;

A2 $$\begin{aligned} \theta (t) = \tan ^{-1}\left( m_c(t) \right) . \end{aligned}$$

The time-average extinction $\overline{I}$ during the steady period of the spray and the ensemble-average $\langle \overline{I} \rangle$ over $i=1$ to *N* repeated sprays are defined as:

A3 $$\begin{aligned} \overline{I}_i = \frac{1}{t_1-t_0} \sum _{t=t_0}^{t=t_1} I_i (x,r,t)\end{aligned}$$

A4 $$\begin{aligned} \langle I \rangle = \frac{1}{N} \sum _{i=1}^{i=N} I_i (x,r,t). \end{aligned}$$

These can be defined to create the time-average, ensemble-average mean extinction profile from which we have defined the spray width and volumetric extinction profiles in Figs. 5-6);

A5 $$\begin{aligned} \langle \overline{I} \rangle = \frac{1}{N \left( t_1-t_0 \right) } \sum _{i=1}^{i=N} \sum _{t=t_0}^{t=t_1} I_i \end{aligned}$$

The temporal stability of the spray is the standard deviation in time ($s_i$) of each individual spray during its steady period, which is then ensemble-averaged to obtain:

A6 $$\begin{aligned} \langle \overline{I}_{stab} \rangle = \frac{1}{N} \sum _{i=1}^{i=N} s_i = \frac{1}{N} \sum _{i=1}^{i=N} \sqrt{ \frac{1}{t_1-t_0} \sum _{t=t_0}^{t=t_1} \left[ I_i - \overline{I}_i \right] ^2} \end{aligned}$$

Similarly, we can define the shot-to-shot repeatability of the spray as the sample standard deviation at each instant in time across *N* repeated measurements ($s_t$), which can then be time-averaged to obtain:

A7 $$\begin{aligned} \langle \overline{I}_{rep} \rangle&= \frac{1}{t_1-t_0} \sum _{t=t_0}^{t=t_1} s_t\nonumber \\  &= \frac{1}{t_1-t_0} \sum _{t=t_0}^{t=t_1} \sqrt{ \frac{1}{N-1} \sum _{i=1}^{i=N}\left[ I_i - \langle I \rangle \right] ^2} . \end{aligned}$$

## Appendix B: Nomenclature

$c_p$: Specific heat capacity
*D*: Orifice exit diameter
*d*: Droplet diameter
$d_{32}$: Droplet Sauter Mean diameter Eq. 7
$d^*$: Theroetical minimum stable droplet size as determined by surface tension Eq. 6
GSD: Geometric standard deviation
$h_{fg}$: Latent heat of vapourisation
*I*: Normalized light extinction in spray
$I_V$: Volume-integrated light extinction in spray Eq. 5
$I_{stab}$: Temporal stability of the normalized light extinction Eq. A6
$I_{rep}$: Shot-to-shot repeatability of the normalized light extinction Eq. A7
$\overline{I}$: Time-average light extinction
$\langle I \rangle$: Ensemble-average light extinction
Ja: Jakob number; ratio of sensible to latent heat Eq. 1
MMAD: Mass mean aerodynamic diameter
*n*: Number of actuations
*N*: Number of repeated trials
*P*: Pressure
$P_{sat}$: Saturated vapor pressure
$P_{amb}$: Ambient (environmental) pressure
*r*: radial distance from orifice
$r_0$: spray centerline radius
Re: Reynolds number ; ratio of inertial to viscous forces
*S*: Droplet surface area
$s_i, s_t$: Sample standard deviation in space and time
*T*: Temperature
$$T_0$$: Ambient temperature
$T_{sat}$: Saturation temperature of the formulation at atmospheric pressure
*t*: time
$t_0, t_1$: start and end time of the steady-state period of the spray
$\overline{U}$: Average (bulk) orifice velocity, flow rate divided by orifice cross-section area
*V*: Droplet volume
We: Weber number ; ratio of intertial to surface tension forces
*x*: axial distance from orifice
$$\rho $$: Density
$\rho _{mix}$: Formulation mixture density at the orifice
$$\mu $$: Dynamic viscosity
$$\sigma $$: Surface tension
