# Supplementary material for: In-vitro Evaluation of Solution Pressurised Metered Dose Inhaler Sprays with Low-GWP Propellants
Source: Pharm Res. 2025 Feb 12;42(2):385–400. doi: 10.1007/s11095-025-03830-6 (PMC11880099; doi:10.1007/s11095-025-03830-6)
Supplement: Supplementary file 1 — (pdf 2000 KB) [file 11095_2025_3830_MOESM1_ESM.pdf]

Supplementary Material for 'In-vitro Evaluation of Solution Pressurised Metered Dose Inhaler Sprays with Low-GWP Propellants'

Daniel J Duke[1], Lingzhe Rao[1], Benjamin Myatt[2],
Phil Cocks[2], Stephen Stein[3], Nirmal Marasini[4], Hui Xin Ong[4,5], Paul Young[4,6]

[1] Laboratory for Turbulence Research in Aerospace & Combustion (LTRAC),
Department of Mechanical & Aerospace Engineering, Monash University, Australia
[2] Kindeva Drug Delivery, Loughborough, United Kingdom
[3] Kindeva Drug Delivery, Woodbury, Minnesota USA
[4] Woolcock Institute of Medical Research, Glebe NSW 2037 Australia
[5] Macquarie Medical School, Faculty of Medicine, Health & Human Sciences,
Macquarie University, Sydney, NSW 2109, Australia
[6] Department of Marketing, Macquarie Business School,
Macquarie University, Sydney, NSW 2109, Australia

Corresponding Author: Dr Daniel Duke (daniel.duke@monash.edu)

1 Average optical extinction measurements

Figure 1 shows the time-average, ensemble-average light extinction for the propellant formulations considered in this study (equation 10). The mean profiles are unremarkable and show relatively little difference. The majority of the differences between propellants are found in the integrated light extinction and higher-order statistics such as temporal stability and shot-to-shot repeatability.

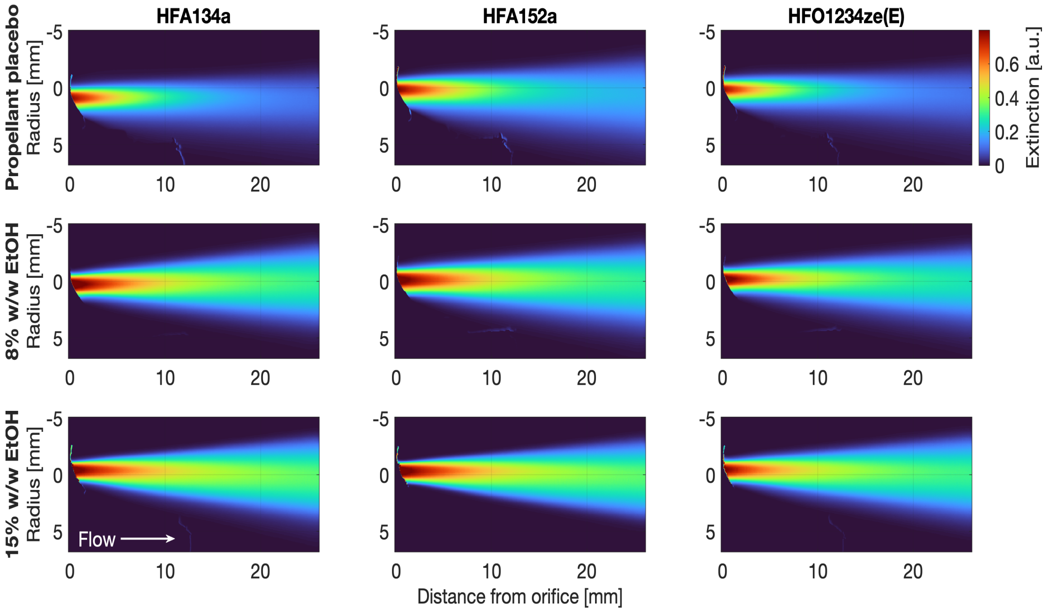

Figure 1: Spatial structure of plumes for all formulations in the near-orifice region for time-average, ensemble-average mean extinction (equation 10). Flow is left-to-right. The horizontal axes are distance from orifice (x) and vertical axes are radius (r), in mm.

2 Volumetric extinction profiles for BDP Solutions

Volumetric extinction profiles $\left\langle \overline{I}_V \right\rangle (x)$ are shown in Figure 6 of the manuscript for propellant-only placebos. The results for BDP solutions follow a similar trend with differences between formulations moderated by the presence of ethanol co-solvent. These are shown in Figure 2. The error bars indicate a combined standard deviation including both temporal and shot-to-shot variation.

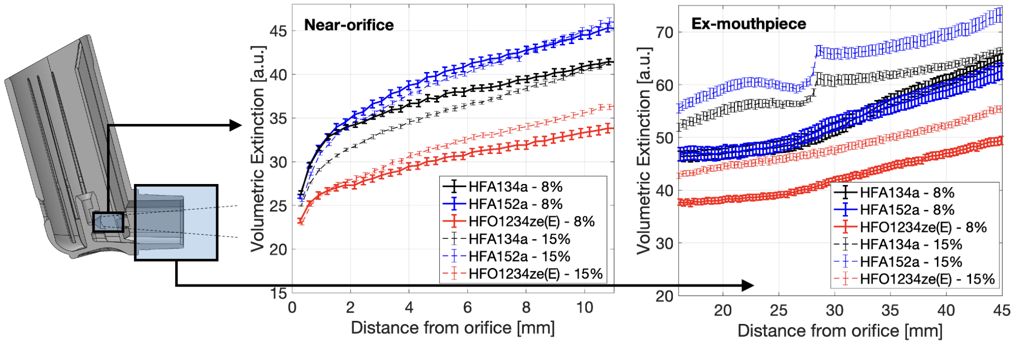

Figure 2: Volumetric extinction profiles $\left\langle \overline{I}_V \right\rangle (x)$ for BDP solution formulations with 8% and 15% w/w ethanol cosolvent.

3 Effect of ethanol on stability and repeatability integrals

Figure 3 shows the effect of adding ethanol on the normalized integrated stability and repeatability as defined in Equations 13–14. The propellant-only placebo is compared to 8% and 15% w/w ethanol solutions of 2.0 mg/mL BDP (indicated by dashed lines). As expected, the profiles flatten and differences are far less marked with increasing ethanol content. As ethanol is added, the spray trends towards the ideal scenario described above where the stability and repeatability are simply described by stretching and scaling of the mean extinction. This is likely due to the greatly reduced volatility of ethanol; flashing, convective mixing and evaporative effects are reduced.

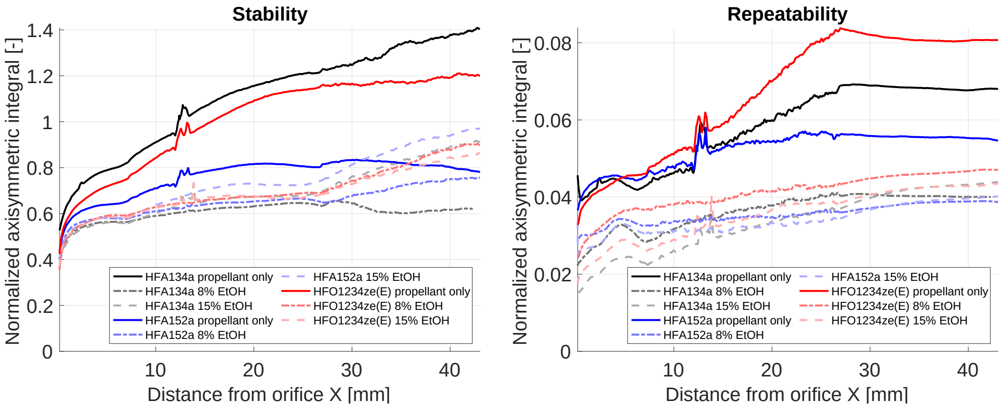

(a) Ratio of radially integrated temporal stability to volumetric extinction $\Psi_{stab}/\left\langle \overline{I}_V \right\rangle$.

(b) Ratio of radially integrated shot-to-shot repeatability to volumetric extinction $\Psi_{rep}/\left\langle \overline{I}_V \right\rangle$.

Figure 3: Radially integrated volumetric profiles for propellant-only placebos, 8% and 15% w/w ethanol solution formulations of 2.0 mg/mL BDP.
